# Supplementary material for: Development of a computer-aided design software for the quantitative evaluation of aesthetic damage
Source: PLoS One. 2019 Dec 18;14(12):e0226322. doi: 10.1371/journal.pone.0226322 (PMC6919621; doi:10.1371/journal.pone.0226322)
Supplement: S1 Code site — 1. (ZIP) [file pone.0226322.s001.zip › [EESC_jr][Projeto]Relatorio_07_21/reports/templates_zip/gentelella-master/vendors/Flot/examples/visitors/index.html]

Flot Examples: Visitors


## Visitors

This plot shows visitors per day to the Flot homepage, with weekends colored.

The smaller plot is linked to the main plot, so it acts as an overview. Try dragging a selection on either plot, and watch the behavior of the other.

Copyright © 2007 - 2014 IOLA and Ole Laursen
